# Supplementary figures and images for: A study of diel and seasonal patterns of loss of commercial lychee fruits to vertebrate frugivores: implications for mitigating a human-wildlife conflict
Source: PeerJ. 2025 Apr 21;13:e19269. doi: 10.7717/peerj.19269 (PMC12020736; doi:10.7717/peerj.19269)

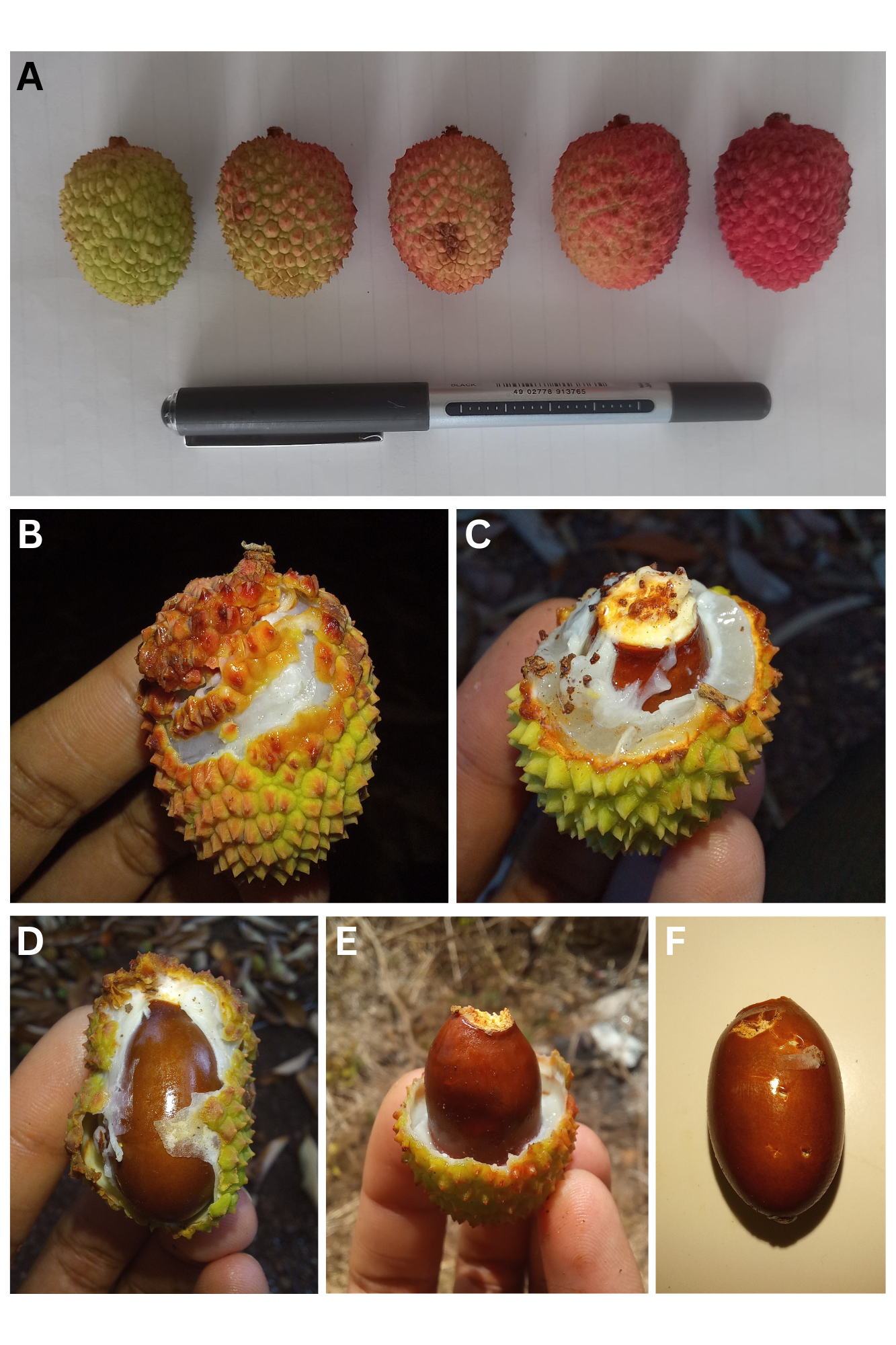

Supplement: Supplemental Information 1 — Lychee at different ripeness stages and different categories of amount of lychee pulp eaten by flying foxes and birds. (A) Lychee ripeness from unripe (left; 0% ripe) to fully ripe (right; 100% ripe). (B) Bite marks on lychee showing that the fruit was damaged but not eaten (0% pulp eaten). (C) >0%–25% pulp eaten. (D) >25%–50% pulp eaten. (E) >50%–75% pulp eaten. (F) Fully eaten (>75%–100%). [file peerj-13-19269-s001.png]
